# Supplementary material for: Mitochondrial Dysfunction in Spinocerebellar Ataxia Type 3 Is Linked to VDAC1 Deubiquitination
Source: Int J Mol Sci. 2022 May 25;23(11):5933. doi: 10.3390/ijms23115933 (PMC9180688; doi:10.3390/ijms23115933)

Figure 1

- **On all membranes, protein bands were visualized using enhanced chemiluminescence method by exposure to Hyperfilm ECL.**

Figure 1C, detected with mouse anti-OPA1, 1:1,000

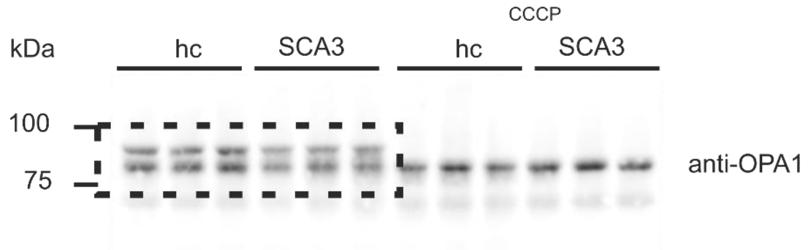

Figure 1C, detected with mouse anti-ACTB for OPA1 blot, 1:5,000

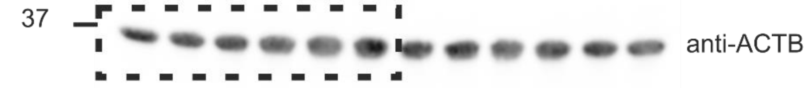

Figure 1C, detected with rabbit anti-MFN2, 1:2,000

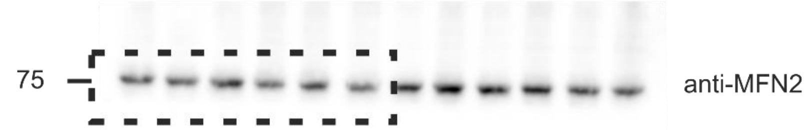

Figure 1C, detected with mouse anti-ACTB for MFN2 blot, 1:5,000

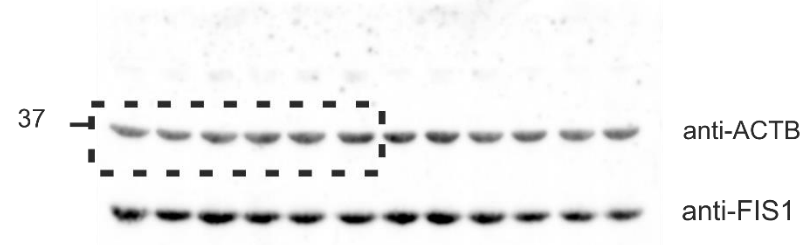

Figure 1C, detected with rabbit anti-FIS1, 1:2,000

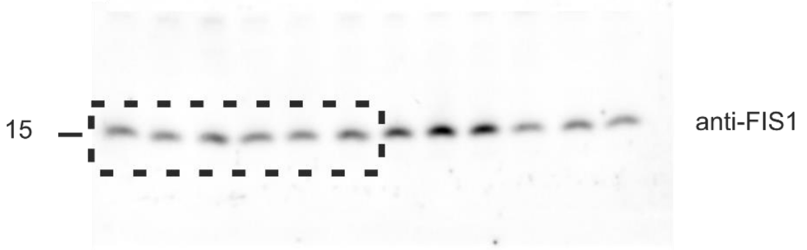

Figure 1C, detected with mouse anti-TUBA for FIS1 blot, 1:5,000

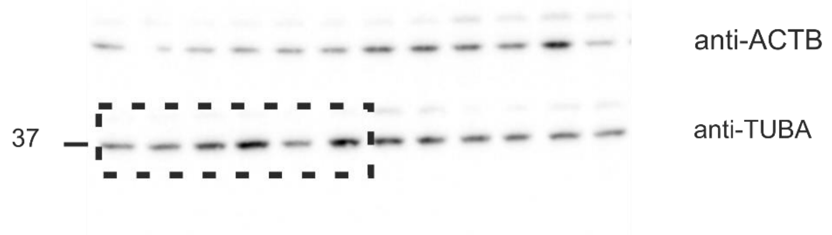

Figure 2:

- **On all membranes, protein bands were visualized using enhanced chemiluminescence method by exposure to Hyperfilm ECL.**

Figure 2D, detected with mouse anti-OXPHOS, 1:1,000

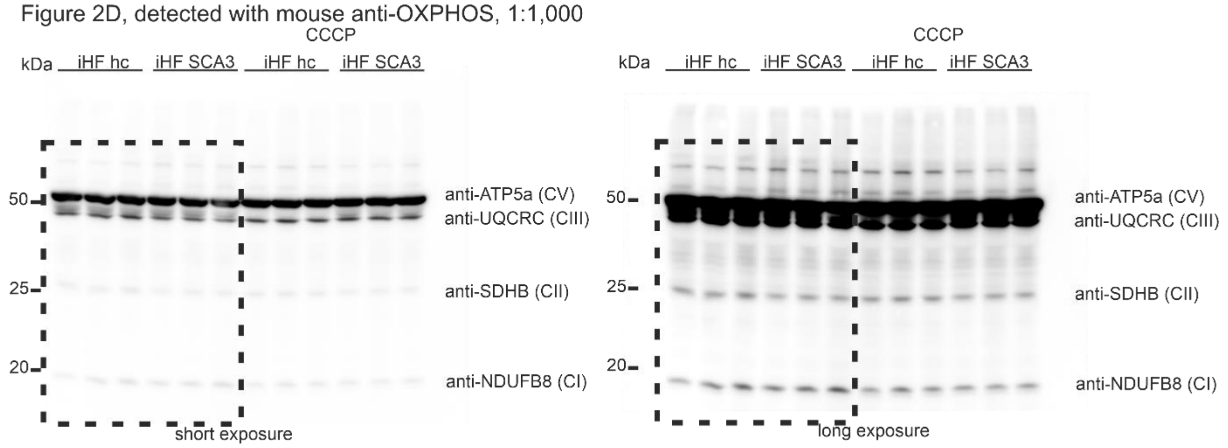

Figure 2D, detected with mouse anti-ACTB, 1:5,000

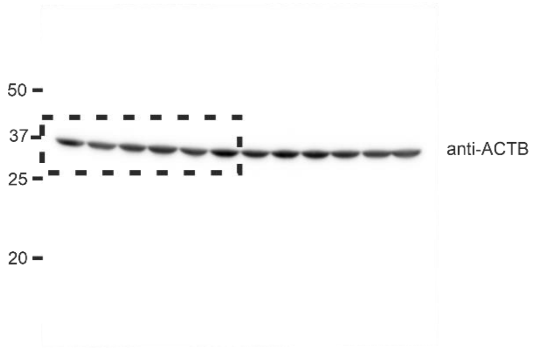

Figure 3:

- **Control = positive control for parkin expression**
- **On all membranes, fluorescence signals were visualized by Licor Fc Odyssey.**

Figure 3A, detected with rabbit anti-CS, 1:1,000

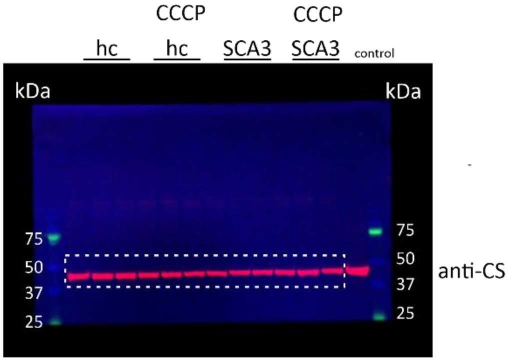

Figure 3A, detected with rabbit anti-parkin, 1:200

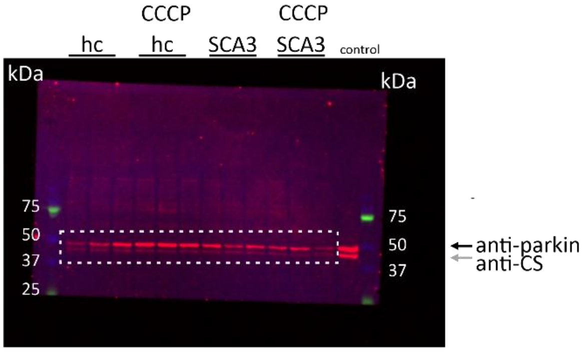

Figure 3A, detected with mouse anti-ACTB, 1:10,000

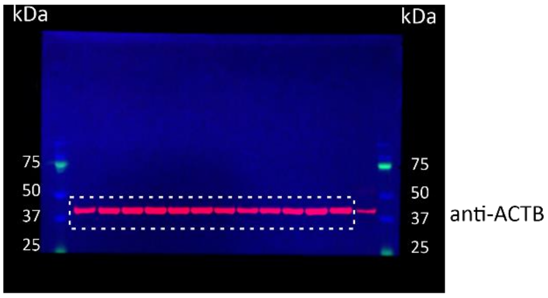

Figure 3A, detected with mouse anti-Pink1, 1:500

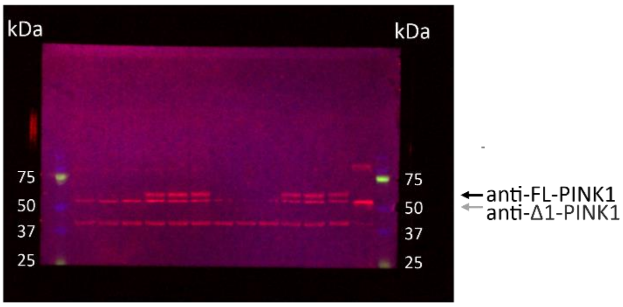

Figure 3A, detected with mouse anti-TOM20, 1:5,000

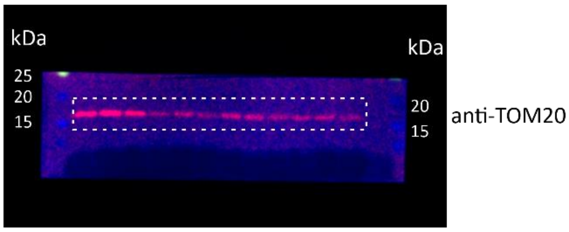

Figure 4:

- **On all membranes, protein bands were visualized using enhanced chemiluminescence method by exposure to Hyperfilm ECL.**

Figure 4A, detected with rabbit anti-VDAC1, 1:10,000

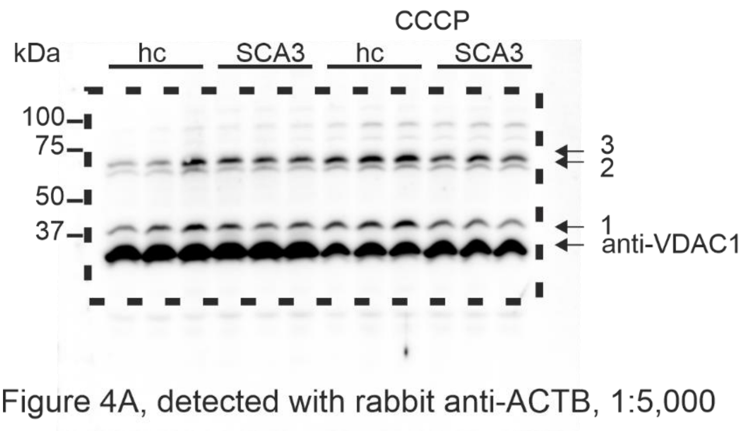

Figure 4A, detected with rabbit anti-ACTB, 1:5,000

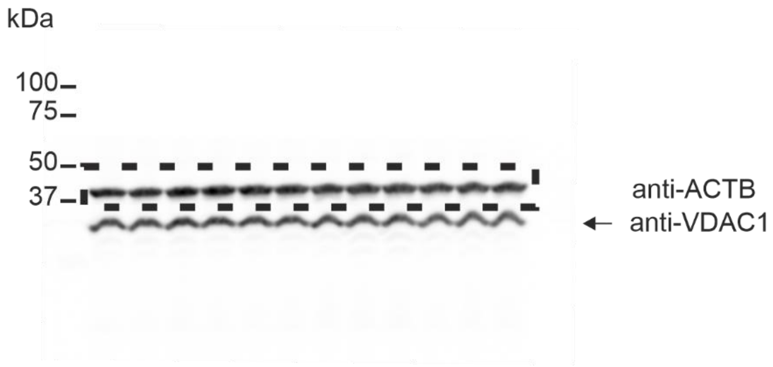

Figure 5

- **On all membranes, fluorescence signals were visualized by Licor Fc Odyssey.**

Figure 5A, detected with rabbit anti-VDAC1, 1:10,000

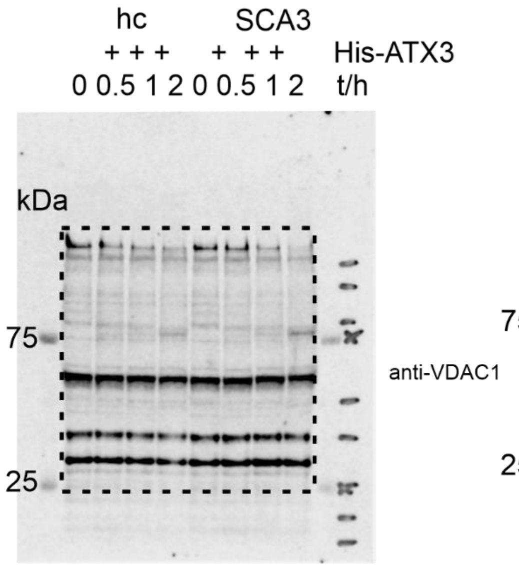

Figure 5E, detected with mouse anti-p53, 1:500

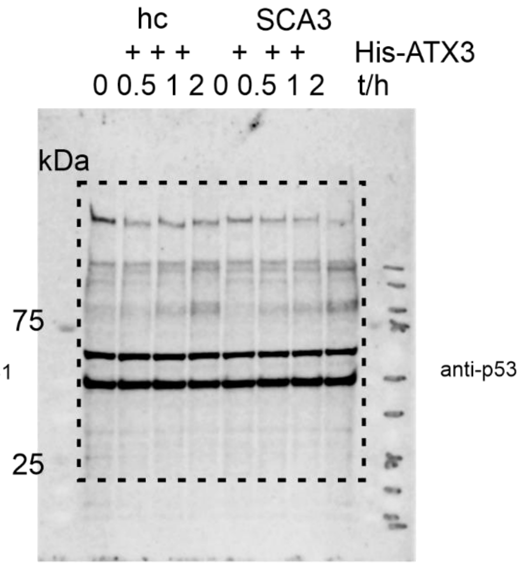

Figure 5F, detected with rabbit anti-K63-pUb, 1:500

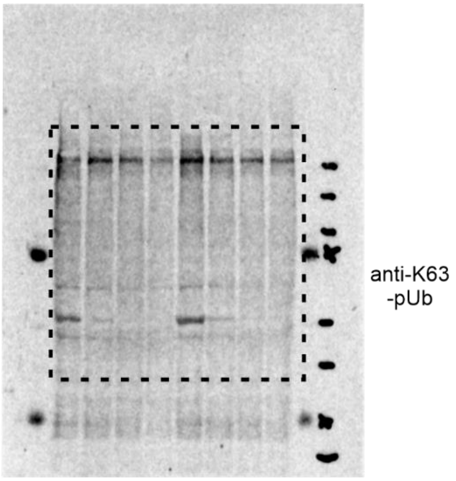

Figure 5G, Ponceau staining

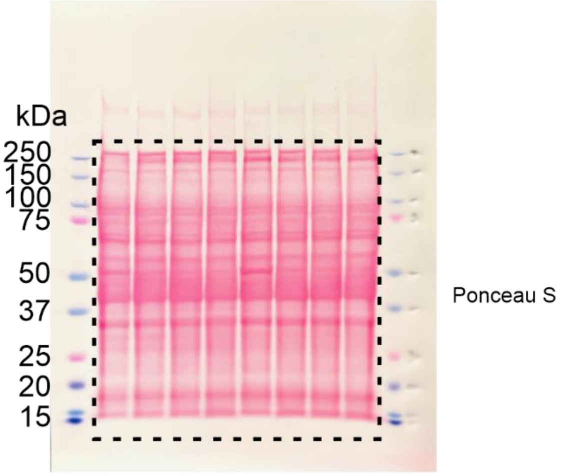

Figure 5F, detected with mouse anti-ATXN3, 1:4,000

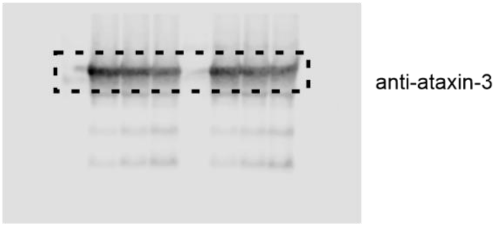

Figure 5F, detected with mouse anti-GAPDH, 1:2,000

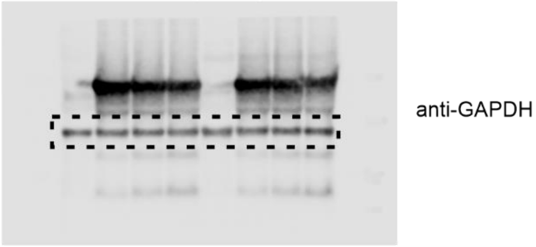

Figure 6

- **On all membranes, protein bands were visualized using enhanced chemiluminescence method by exposure to Hyperfilm ECL.**

Figure 6A, detected with rabbit anti-p62, 1:1,000

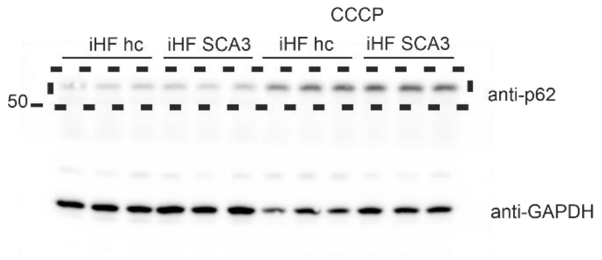

Figure 6A, detected with mouse anti-LC3, 1:100

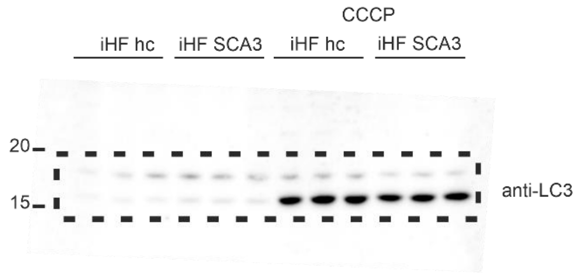

Figure 6A, detected with mouse anti-TUBA, 1:5,000

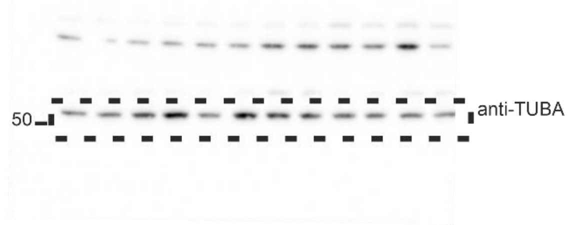

Figure 6A, detected with mouse anti-GAPDH, 1:2,000

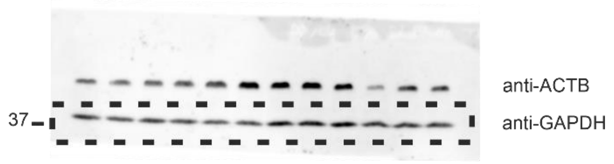

Figure 8:

- **For all membranes, fluorescence signals were visualized by Licor Fc Odyssey.**

Figure 8A, detected with rabbit anti-parkin, 1:200

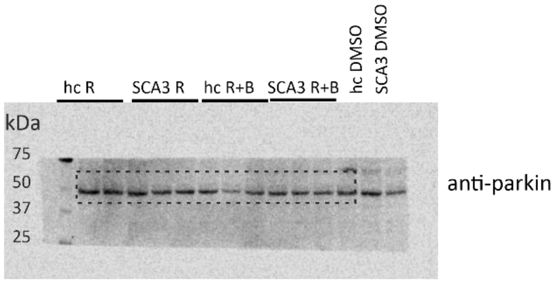

Figure 8A, detected with mouse anti-Pink1, 1:500

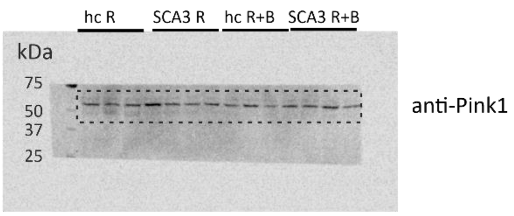

Figure 8A, detected with mouse anti-ACTB 1:10,000

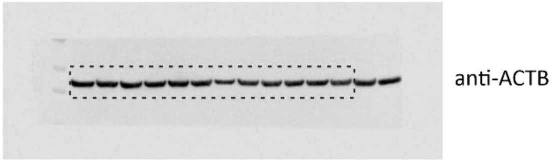

Figure 8A, detected with rabbit anti-p62, 1:1,000

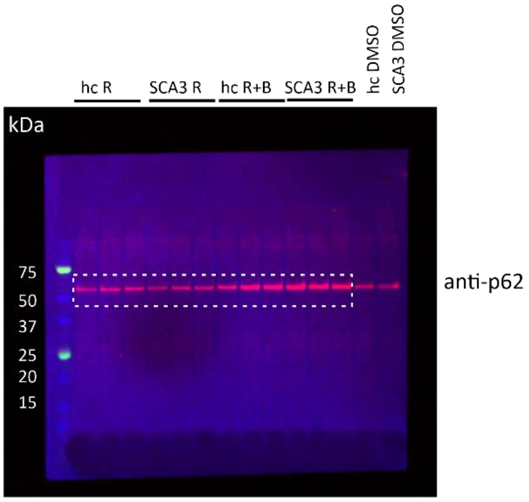

Figure 8A, detected with rabbit anti-CA, 1:1,000

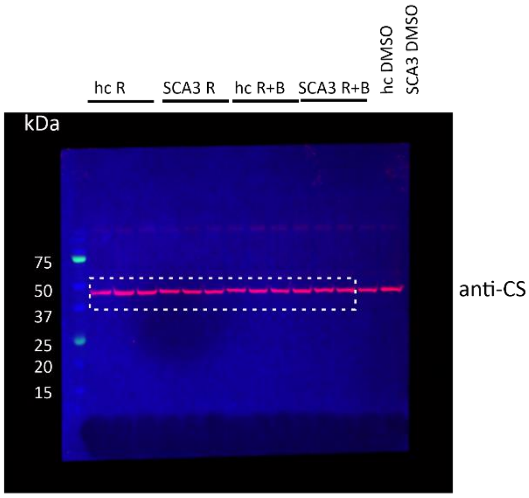

Figure 8A, detected with mouse anti-LC3, 1:100

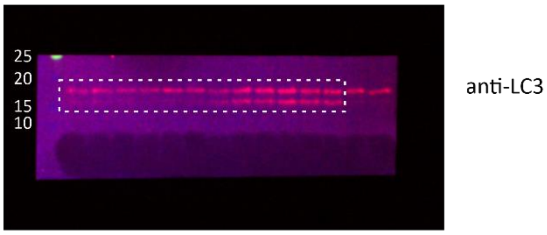

Figure 8A, detected with mouse anti-ACTB, 1:10,000

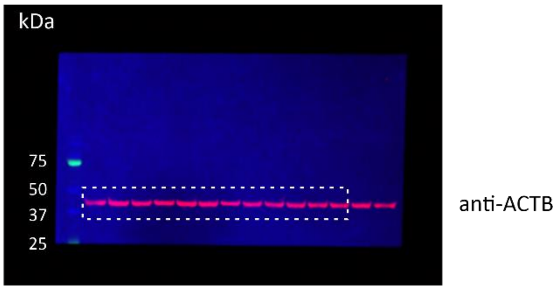

Figure A3: Supplement

- **For VDAC and the respective ACTB, protein bands were visualized using enhanced chemiluminescence method by exposure to Hyperfilm ECL. CS, parkin, PINK1 and the respective ACTB, fluorescence signals were visualized by Licor Fc Odyssey.**

Figure A3A Supplement, detected with rabbit anti-CS, 1:1,000

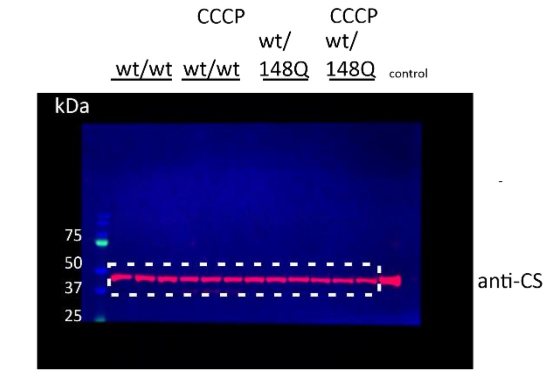

Figure A3A Supplement, detected with rabbit anti-parkin, 1:200

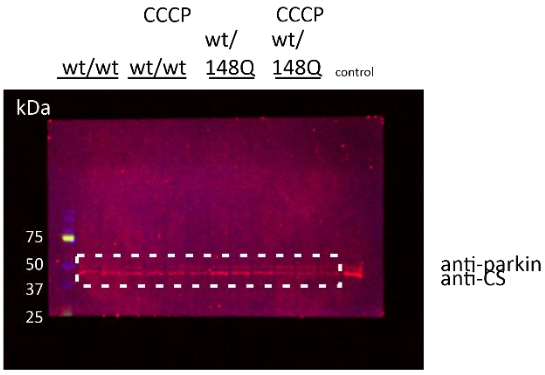

Figure A3A Supplement, detected with mouse anti-ACTB, 1:10,000

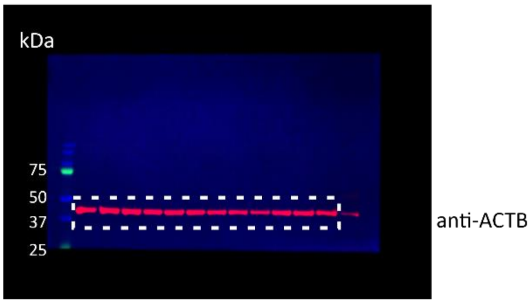

Figure A3A Supplement, detected with mouse anti-parkin, 1:500

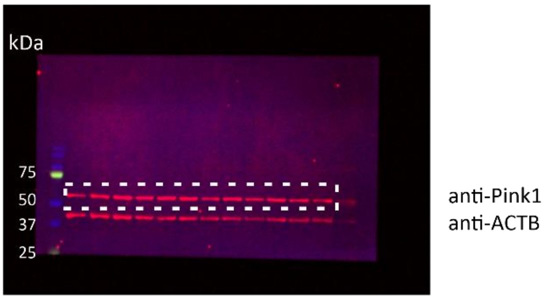

Figure A3D Supplement, detected with rabbit anti-VDAC1, 1:10,000

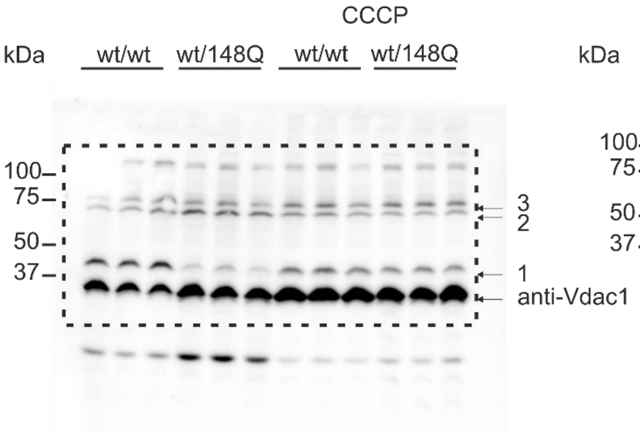

Figure A3D Supplement, detected with mouse anti-ACTB, 1:5,000

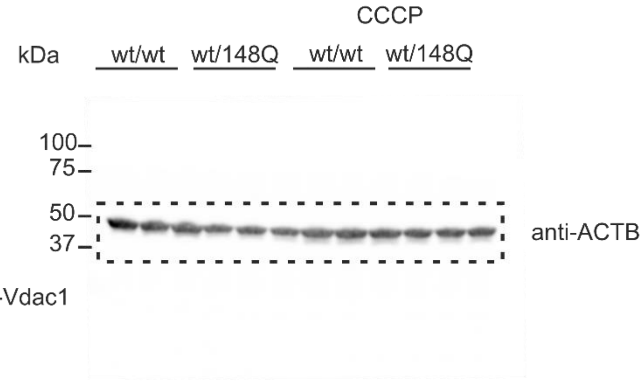

Supplement: Supplementary file 1 [file ijms-23-05933-s001.zip › ijms-1683154-raw Western Blots.pdf]
