# Supplementary material for: Mitochondrial Dysfunction in Spinocerebellar Ataxia Type 3 Is Linked to VDAC1 Deubiquitination
Source: Int J Mol Sci. 2022 May 25;23(11):5933. doi: 10.3390/ijms23115933 (PMC9180688; doi:10.3390/ijms23115933)
Supplement: Supplementary file 1 [file ijms-23-05933-s001.zip › ijms-1683154-supplementary.pdf]

Supplementary materials

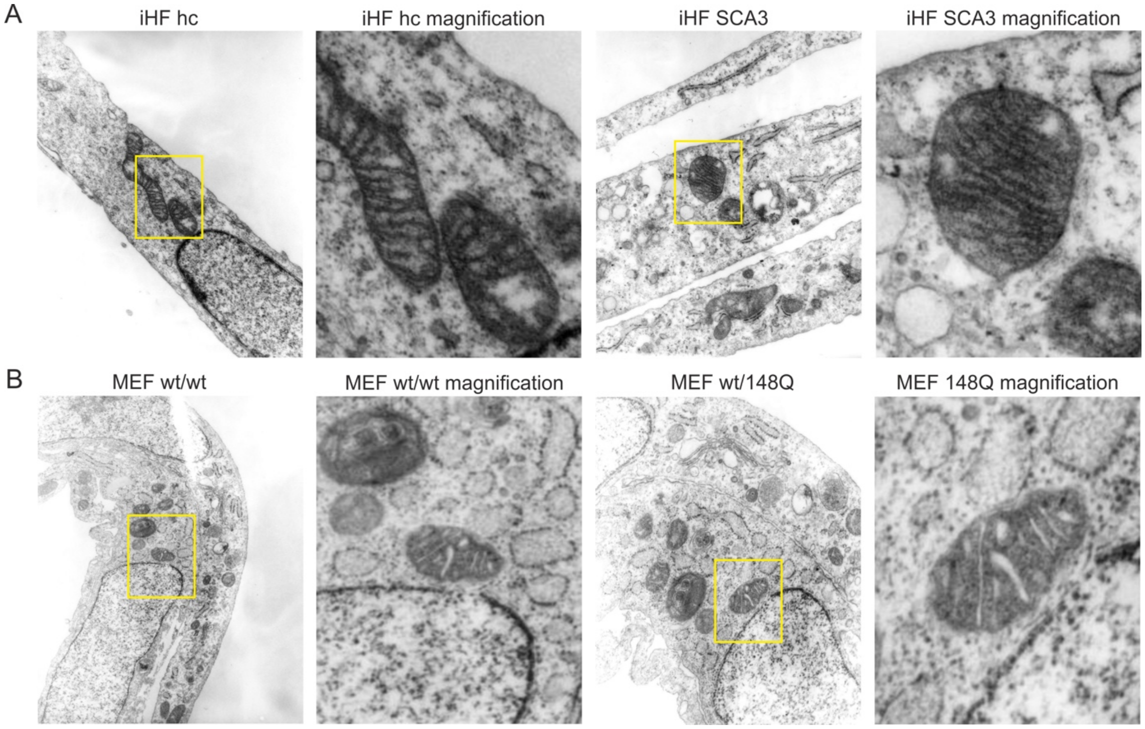

Supplementary Figure S1. Mitochondrial cristae structure is intact in fibroblasts from different cells expressing polyQ-expanded ataxin-3. **(A)** Electron microscopy pictures of SCA3 patient-derived (iHF SCA3) and control fibroblasts (iHF hc) and (**B**) in MEF isolated from SCA3 transgenic mice expressing ataxin-3 148Q (MEF 148Q) and wildtype controls (MEF wt/wt). hc = healthy control, SCA3 = fibroblasts derived from patients, iHF = immortalized human fibroblasts, MEF = mouse embryonic fibroblasts, wt = wildtype.

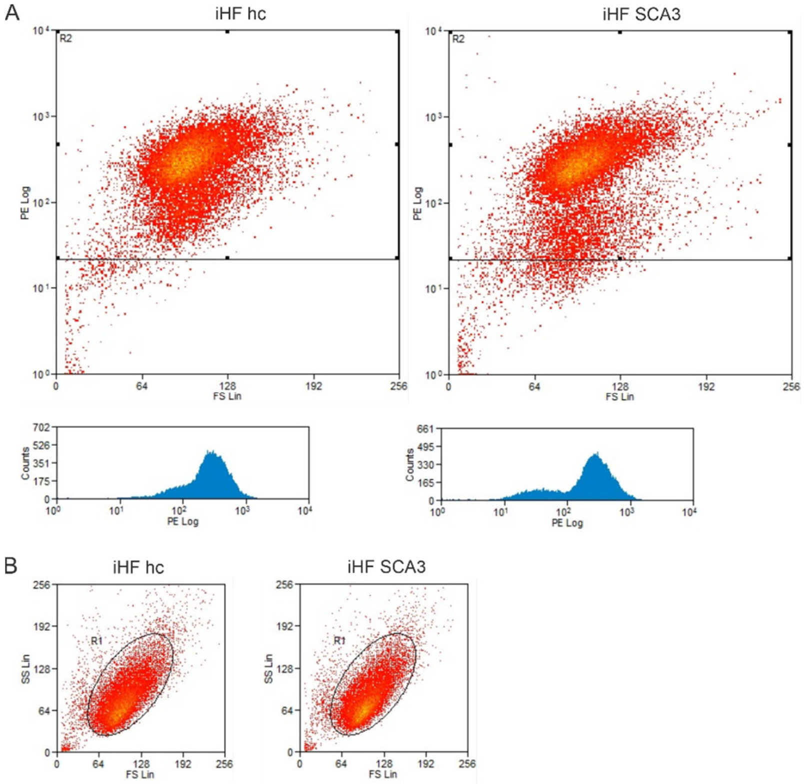

Supplementary figure S2. FACS analyses of mitochondrial membrane potential in SCA3 disease conditions. Mitochondrial membrane potential was measured by TMRE staining and subsequent FACS analyses. **(A)** Signal intensity (PE) was plotted against FS Lin (A, upper plot) or counts (**A**, lower plot). **(B)** Plotting SS Lin against FS Lin did not reveal any differences between both genotypes. hc = healthy control, SCA3 = fibroblasts derived from SCA3 patients, iHF = immortalized human fibroblasts.

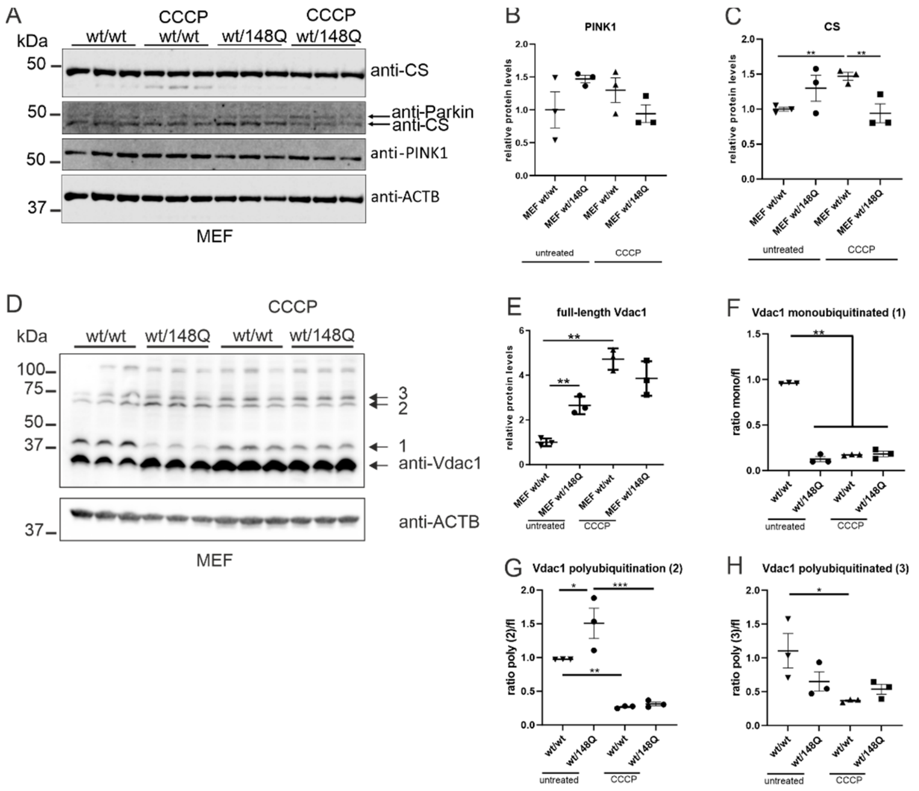

Supplementary Figure S3. PolyQ-expanded ataxin-3 modulates VDAC1 ubiquitination in MEFs**. (A)** Western blot analyses of citrate synthase (CS), PINK1 and parkin in MEF 148Q compared to wildtype controls. Beta-Actin (ACTB) is shown as loading control. (B, C) Statistical analyses were performed by one-way ANOVA and Turkey´s post-test. Genotypes and treatments were normalized to untreated MEF wt/wt. **(D)** Protein level of full-length VDAC1 and ubiquitinated form of VDAC1 were analyzed by Western blot. Beta-Actin (ACTB) is shown as loading control. (E-H) For statistical analyses, genotypes and treatments were normalized to untreated MEF wt/wt and significant differences evaluated by one-way ANOVA. * $P < 0.05$, ** $p < 0.01$, *** $p < 0.001$. Values are shown as mean +/– SEM. N = 3. MEF = mouse embryonic fibroblasts, wt = wildtype.

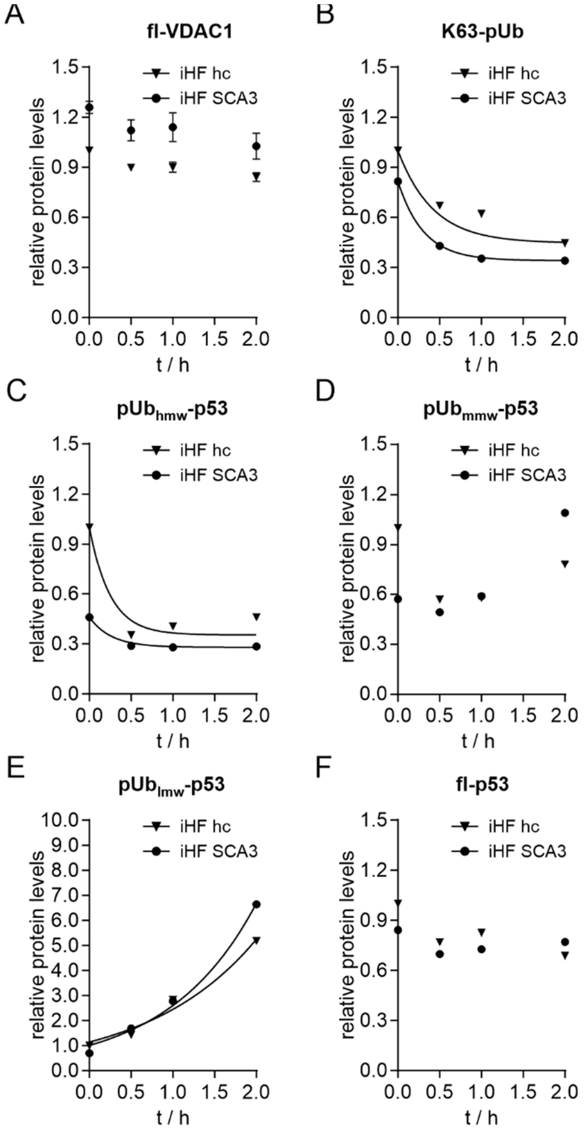

Supplementary figure S4. Quantitative analysis of ataxin-3 substrates as detected in DUB assay *in vitro*. (**A**) Quantitative analysis of full-length (fl) VDAC levels in cell extracts of immortalized human fibroblasts from healthy controls (iHF hc) or SCA3 patients (iHF SCA3) incubated with purified His_6-ataxin-3 for up to 2 h. (**B**) Quantitative analysis of K63-linked polyubiquitin (pUb) levels in the same samples, showing their time-dependent reduction. N = 1. (**C-F**) Quantitative analysis of high-molecular weight (hmw, **C**), medium-molecular weight (mmw, **D**), and low-molecular weight (lmw, **E**) polyubiquitin (pUb) p53 levels, and of full-length (fl, **F**) p53 levels in the same samples. N = 1. Curves for pUb_{hmw}-p53 were extrapolated based on a one-phase decay nonlinear regression model, whereas curves for pUb_{lmw}-p53 were extrapolated using an exponential growth equation.

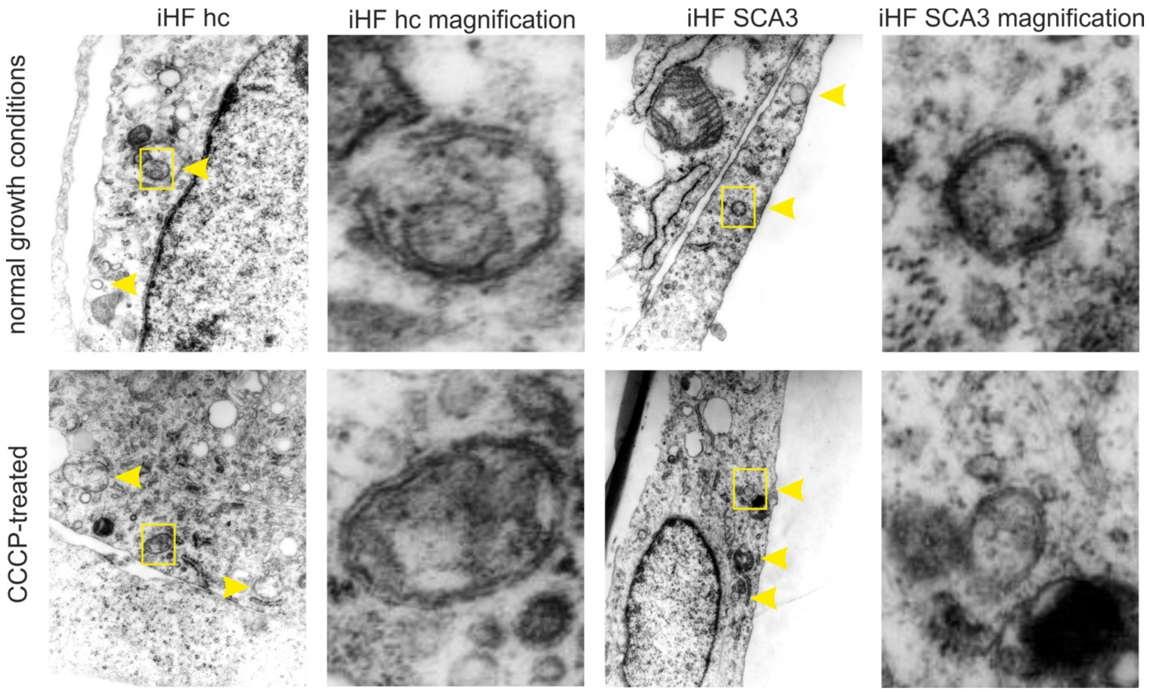

Supplementary figure S5. Evaluation of autophagosomes in SCA3 patient fibroblasts using electron microscopy. Fibroblasts from SCA3 patients and healthy controls were either grown under normal conditions or upon CCCP treatment (6h) and autophagosomes were examined by electron microscopy. Autophagosomes display a round shape and a double membrane. Yellow arrow heads point to organelles matching these criteria. Magnifications show that cargo of autophagosomes cannot be clearly identified in this experiment. hc = healthy control, SCA3 = fibroblasts derived from patients, iHF = immortalized human fibroblasts.
